# REHABILITATION IN GAZA: A CALL TO THE REHABILITATION COMMUNITY AND TO THE EUROPEAN UNION OF MEDICAL SPECIALISTS (UEMS)

**DOI:** 10.2340/jrm.v58.46725

**Published:** 2026-09-02

**Authors:** Paul CARROLL, Fahim ANWAR, Christoph GUTENBRUNNER, Karol HORNÁČEK, Ingebjørg IRGENS, Carlotte KIEKENS, Jorge LAÍNS, Thierry LEJEUNE, Stefano NEGRINI, Eirik VIKANE, Maria Gabriella CERAVOLO

**Affiliations:** 1 National Rehabilitation Hospital, Dún Laoghaire, Ireland; 2 Cambridge University Hospital NHS Foundation Trust, Cambridge, UK; 3 Department of Rehabilitation and Sports Medicine, Hannover Medical School, Hannover, Germany; 4 Slovak Medical University and University Hospital, Bratislava, Slovakia; 5 VID Specialised University, Oslo, Norway; 6 Laboratory of Evidence-based Medicine, IRCCS Galeazzi S. Ambrogio Hospital, Milan, Italy; 7 Centro de Medicina de Reabilitação da Região Centro-Rovisco Pais, Tocha, Portugal; 8 IREC, Institute of Experimental and Clinical Research, UCLouvain, Brussels; 9 Physical Medicine and Rehabilitation department, Cliniques Universitaires Saint-Luc, Brussels, Belgium; 10 Department of Biomedical, Surgical and Dental Sciences, University of Milan, Milan, Italy; 11 Department of Physical Medicine and Rehabilitation, Haukeland University Hospital, University of Bergen, Bergen, Norway; 12 Department of Experimental and Clinical Medicine, Marche Polytechnic University, Ancona, Italy

This commentary seeks to raise awareness of the rehabilitation needs arising from the conflict in Gaza and to call for urgent action by the international rehabilitation community.

While its humanitarian consequences have been widely reported, the scale and long-term burden of conflict-related disability have received far less attention. By 27 June 2026, 173,480 people had been injured, and the World Health Organization (WHO) estimates that 43,370 of them – 1 in 4 of the injured, 2% of the pre-conflict population – have potentially life-changing injuries needing specialized rehabilitation for years (1, 2).

The magnitude of the suffering, the political complexity, and the length of the conflict may foster helplessness and discourage engagement. However, rehabilitation has always been founded on the belief that functioning, participation, and quality of life can be improved even in the most challenging circumstances. The rehabilitation community, professional organizations, and individual clinicians therefore all have a role in advocating for access, rebuilding capacity, and showing solidarity with colleagues in Gaza.

## CONTEXT

The Gaza Strip, home before the conflict to some 2.1 million people (> 1 million below age 18), is occupied territory: Israel controls the sea, the air, and what crosses its borders, so healthcare there has always depended on permission for patients, staff, equipment, and supplies to cross (3). After the Hamas attacks on Israel of 7 October 2023, in which more than 1,200 people were killed, Israel began large-scale military operations; the number of Gazans killed approaches 80,000, considered an under-report by at least 40% (4).

## EMERGING REHABILITATION NEEDS

The principal mechanism of injury is blast (1), unlike the leading causes globally, falls and road trauma (5). Many have injuries at multiple sites (6), polytrauma making rehabilitation longer and more team-dependent. Injuries comprise major limb injury including amputation, traumatic brain and spinal cord injury, burns, and severe maxillofacial, ocular, and peripheral nerve injury (Table I). Needs also arise from chronic disease and pre-existing disability, compounded by displacement and interrupted care, and from a rise in Guillain–Barré syndrome (1, 7).

**Table I:** Type and estimated number of major injuries requiring rehabilitation in Gaza, 7 October 2023–1 April 2026

Type of major injury	% of all injuries	Estimated number (0–30% polytrauma)
Major extremity injury	13	22,366–29,075
Other major injury*	5	8,602–11,183
Limb amputation	3	5,161–6,710
Major burn	2	3,441–4,473
Spinal cord injury	1.2	2,065–2,684
Major traumatic brain injury	0.8	1,376–1,789
Total	25	43,011–55,914

* Includes pelvic, thoracic, abdominal, and maxillofacial injuries. Percentages are based on the 172,043 people reported injured as of 1 April 2026; ranges reflect assumed polytrauma rates of 0–30%. WHO’s preferred estimate of injuries requiring extended rehabilitation (15–30% polytrauma) is 49,462–55,914. An estimated 43,011 people – 2% of the pre-conflict population, up to a quarter of them children – have potentially life-changing injuries. Source: WHO (1).

Two statistics underscore the magnitude of the crisis: since October 2023, more than 5,000 amputations have been performed, with children accounting for 1 in 5 amputees (1). UNICEF also reports that Gaza has the world’s highest per-capita rate of childhood amputation (7). For every child fitted with a prosthesis, decades of replacement, maintenance, and rehabilitation will be required. WHO estimates 2,065–2,684 spinal cord injuries (1); Ireland, with twice Gaza’s population and an intact health system, sustains about 60 annually (8) – 35 to 45 years of Irish incidence in 2½ years. Mental health needs are high and often concomitant with severe injury, with children especially vulnerable (9, 10). Malnutrition compounds every injury: fractures that would normally unite in 6 weeks have remained unhealed after 6 months for want of protein, and evidence from historical famines indicates that survivors carry chronic disease and impairment for decades (7, 11).

## REHABILITATION CAPACITY

Capacity has moved the opposite way. Fifty rehabilitation professionals have been killed since October 2023 (1). No rehabilitation facility in Gaza is fully functional; only 2 offer integrated physiotherapy, occupational therapy, and psychosocial support (7). Over 400 patients await specialized beds, and those admitted are often discharged early into tents, with 92% of housing destroyed (1, 7). With so few beds, services prioritize spinal cord and brain injury, reducing access for other health conditions (1), similarly to what happened in the global emergency during the COVID pandemic (12).

### The ceasefire has not restored access

The most consequential finding in the WHO’s May 2026 update is not a casualty figure. It is that no equipment for rehabilitation facilities entered Gaza between May 2024 and 14 April 2026 – almost 2 years, including throughout the ceasefire – while entry of assistive products remained severely constrained. Eighteen shipments were then pending, waiting an average of 136 days and up to 526 days; 4 consignments of assistive products had been rejected outright.

The conventional sequence – end the fighting, then rebuild – assumes hostilities are what prevent rehabilitation. Gaza shows otherwise. The decisive constraint is administrative: what may cross a border, and how long it waits. A ceasefire alone cannot restore rehabilitation services. Without unimpeded access to assistive products, prosthetic components, equipment, and fuel, rehabilitation cannot be delivered. Without rehabilitation, survival is too often followed by preventable complications, avoidable dependence, and lifelong exclusion from education, employment, and community life.

Advocacy should therefore be specific: “reconstruction” is too diffuse to matter to a child waiting for a prosthetic socket. The evidence points to 4 urgent priorities: clearance of the pending shipments; an end to the rejection of assistive products as a category; a guaranteed fuel allocation for outreach, reduced in 2025 to operating on foot (7); and functioning medical evacuation pathways.

Since the ceasefire, there are signs of limited improvement: some goods are entering, and some patients are being evacuated to Egypt and Jordan, though not as a continuous flow, and Israel is not currently receiving patients as it did before the conflict (13). These gains are fragile and far below what Table I implies.

### Why rehabilitation is different, and what must be rebuilt

Emergency interventions save lives and reduce disability; rehabilitation restores lives and prevents the complications that end them. For brain and spinal cord injury, major limb loss, severe burns, and complex musculoskeletal trauma, it is not a short-term intervention but a continuum of specialized care over months, years, and often a lifetime – a need extending far beyond the cessation of hostilities and exceeding any single health system.

What must be rebuilt is specified in the Minimum Rehabilitation Service Package for Gaza (14, 15). For Europe, action could include:

- *Advocacy:* placing Gaza on the formal agendas of national and European rehabilitation organizations, with named responsibility.
- *Education and workforce rebuilding:* the universities that trained Gaza’s rehabilitation professionals have been destroyed, and training is now partial, delivered in hospitals or online against limited internet access and scarce computers (7). Training for multiprofessional partnerships in prosthetics and orthotics, spinal cord and brain injury, and paediatric rehabilitation is needed, alongside rebuilding academic capacity.
- *Telehealth and volunteering:* structured remote clinical support and case supervision; physical travel will remain possible for a few.
- *Structural collaboration:* cross-border referral, funding, and travel for patients needing specialized rehabilitation abroad, and joint research.

### Professional responsibility and a call to action

Rehabilitation carries an ethical commitment of its own: The White Book on Physical and Rehabilitation Medicine in Europe grounds the speciality in equity and the rights of persons with disabilities, recognizing rehabilitation not as a charitable service but as a fundamental human right (16). Professional organizations exist not only to advance knowledge and clinical excellence but also to defend the ethical foundations of practice; silence in the face of humanitarian need on this scale reduces their specific role. The response has been slow, and professional medical bodies have not been among the faster voices (17, 18). Whatever gains have been achieved are the result of the largely unseen humanitarian and clinical community already on the ground, committed not to political interests but to the humanitarian imperative that care be provided solely based on need. The European Union of Medical Specialists (UEMS) promotes the highest standards of specialist practice, advocates for conditions enabling optimal patient care, and upholds high standards of medical ethics (19).

Our argument rests on 2 things only: documented rehabilitation needs, and obligations already established in international law. What counts is who needs rehabilitation, and who is best placed to provide it.

Israel participates in the UEMS through the Israeli Medical Association as an Associate Member. Israel possesses internationally recognized rehabilitation expertise and is the closest country able to provide highly specialized rehabilitation to people in Gaza. As the occupying power, Israel also carries obligations in law.

Article 55 of the Fourth Geneva Convention obliges an occupying power, to the fullest extent of the means available to it, to ensure the population’s medical supplies and to bring in “medical stores and other articles” where local resources are inadequate. Article 56 obliges it to maintain the medical services of the occupied territory (20). Assistive products and prosthetic components are such stores, and the qualifying phrase sets a demanding standard of due diligence, not an escape clause (21). Article 59 speaks directly to the shipments above: where a population is inadequately supplied, the occupying power “shall agree to relief schemes … and shall facilitate them by all the means at its disposal”, including medical supplies (20) – a duty read as absolute, admitting no ground for blocking such relief (21).

The rehabilitation community should ask more of its institutions than expressions of concern. We call upon the UEMS Physical and Rehabilitation Medicine (PRM) Section to urge the UEMS to recognize the rehabilitation crisis in Gaza as directly relevant to the profession and to engage formally with its Associate Member, Israel, requesting a public commitment to advocate for humanitarian access to rehabilitation supplies and to support the transfer of patients.

Access to rehabilitation should be determined by clinical need and human dignity, not nationality or political circumstance. Others have already acted: the British Medical Association (BMA) suspended ties with the Israeli Medical Association in June 2025, after which the BMA appealed for medical supplies to be let through; the South African Medical Association followed in October 2025; and a petition of over 1,150 health professionals seeks its suspension from the World Medical Association, which opposes excluding members for their governments’ actions (22). Our proposal is narrower – engagement first, on rehabilitation access – and only if that is refused should the UEMS consider whether continued Associate Membership remains compatible with the ethical principles expected within its community, not as a political sanction but as an affirmation that membership entails responsibilities as well as privileges. The profession is judged not only by the care it provides but by its willingness to defend patients when access to care is threatened.

## CONCLUSION

Rehabilitation will not undo what has happened in Gaza. Still, it decides whether tens of thousands of people, a quarter of them children, live dependent or take part in rebuilding their society. The needs are documented, the service package is defined, and the obligations are set out in law. What is missing is access, a workforce and sustained professional commitment. The first 2 depend on decisions others must take; the third is ours.
